# Correlates of Myopathy in Diabetic Patients Taking Statins

**DOI:** 10.7759/cureus.37708

**Published:** 2023-04-17

**Authors:** Sara Tariq, Lakshmi Goriparthi, Dina Ismail, Gauvain Kankeu Tonpouwo, Milan Thapa, Khizer Khalid, Ayden Charlene Cooper, Gutteridge Jean-Charles

**Affiliations:** 1 Internal Medicine, Mayo Hospital, Lahore, PAK; 2 Internal Medicine, JC (Jean-Charles) Medical Center, Orlando, USA; 3 General Surgery, Osmania Medical College, Hyderabad, IND; 4 Family Medicine, University Hassan II of Casablanca Faculty of Medicine and Pharmacy, Casablanca, MAR; 5 Internal Medicine, Faculty of Medicine, University of Lubumbashi, Plaine Tshombé, Lubumbashi, COD; 6 Internal Medicine, Monmouth Medical Center, Long Branch, USA; 7 Internal Medicine, AdventHealth Orlando Hospital, Orlando, USA

**Keywords:** management of statin-associated myopathy, statin-induced myopathy pathophysiology, statin-induced myopathy, diabetes and cvd, statins, diabetes mellitus

## Abstract

Diabetes is one of the most common chronic ailments; its incidence has reached epidemic proportions in the 21st century. Diabetes significantly increases micro and macrovascular complications, which are effectively managed with statins. Therefore, statins’ pharmacokinetics, pharmacodynamics, and pharmacogenetics have been extensively studied. Although statins act as a keystone in preventing cardiovascular complications, at the same time, they pose a threat to the quality of life of diabetics due to the resulting muscular side effects. This article summarizes the prevalence, clinical manifestations, pathophysiology, and risk factors of statin-induced myopathy in diabetic patients. Among the diverse predisposing risk factors, the primary variables identified for causing myopathy in diabetic patients include age, gender, ethnicity, duration and severity of illness, comorbid conditions, level of physical activity, alcohol use, cholecalciferol (vitamin D3) levels, type and dose of statins, and anti-diabetic drugs or other drugs used concomitantly. In addition, cardiovascular risk quotients also potentially impact diabetic patients making them more vulnerable to developing myopathy from statins. Therefore, this study highlights the importance of managing statin-associated myopathic side effects by providing consensus guidelines on diagnostic, monitoring, and treatment strategies. We also discussed statins’ prognostic value in reducing cardiovascular events in diabetic individuals.

## Introduction and background

Diabetes mellitus (DM) is one of the most serious global health crises of the 21st century. In 2019, diabetes caused an estimated 1.5 million deaths [1]. Cardiovascular disease (CVD) majorly contributes to morbidity and mortality in type 2 DM (T2DM) patients [2]. Patients with T2DM have a risk of death from cardiovascular causes that is two to four times that of persons without DM [3]. A major cause is atherogenic dyslipidemia in T2DM, which consists of elevated triglyceride levels, decreased high-density lipoprotein (HDL), and a higher proportion of smaller and denser low-density lipoprotein (LDL) particles, which are more susceptible to oxidation and may thereby increase the risk of cardiovascular events [4,5]. Optimizing lipid-lowering therapy use among type 2 diabetic patients has been considered one of the beneficial approaches to decreasing the overall cardiovascular disease burden [6]. In T2DM patients, statin treatment is a cornerstone of the physicians' efforts to prevent primary and secondary cardiovascular disease [7]. In the ACC/AHA (American College of Cardiology/American Heart Association) 2013/2019 guidelines, 40-75 years old T2DM patients are one of the four major statin benefit groups to provide CVD primary and secondary prevention [8,9]. American Diabetes Association (ADA) standards of care guide all T2DM patients over 40 years to take moderate-intensity statins as primary prophylaxis. On the other hand, higher-intensity statins are recommended for the secondary prevention of diabetic patients with CVD or increased CVD risks, such as those with abnormal LDL cholesterol (LDL-C) levels, smokers, hypertension, or albuminuria [10].

Although statin use does not frequently cause side effects, it still can result in some debilitating symptoms involving the muscular, gastrointestinal, neural, and hepatic systems of the body [11]. Observational researchers estimate that 10-15% of statin users suffer from statin-related muscular side effects ranging from mild myalgia to rhabdomyolysis with considerable creatine kinase (CK) elevations [12]. Statin-induced myopathy is associated with several drug-related risk factors, such as type, dose, lipophilicity, metabolism, and drug-drug interactions [13]. Several medications, especially cytochrome P450 inhibitors, can potentiate the effect of statin, increasing the susceptibility to musculoskeletal effects [14]. Other factors implicated in statin-related myopathy are patient-specific, such as age, gender, ethnicity, and comorbidities [15]. Statin-associated myopathy is a significant cause of rising statin intolerance and commonly cause discontinuation among patients taking statins [12]. At the same time, stopping statins is considered counter-therapeutic and linked with a high rate of major adverse cardiovascular events (MACE) among older patients suffering from cardiovascular disorders [16]. 

Therefore, identifying more susceptible individuals and understanding the pathophysiology of statin-induced myopathy will help us better manage such adverse incidents and reduce the risk of undesirable statin discontinuation.

## Review

Statins

Definition

Statins are a well-known class of drugs used to treat hypercholesterolemia, also known as 3-hydroxy-3-methylglutaryl coenzyme A (HMG-CoA) reductase inhibitors. It has been shown that they reduce the risk of cardiovascular morbidity and mortality in individuals with coronary artery disease (CAD) or at risk of CAD [17]. In addition, statins are the most prescribed medication due to their consistent clinical effectiveness in lowering LDL cholesterol [18]. 

Pharmacokinetics and Pharmacodynamics

Statins reduce cholesterol in the liver by inhibiting the enzyme HMG-CoA reductase, which converts HMG-CoA to mevalonate, the rate-limiting step in cholesterol synthesis. It causes overexpression of LDL receptors in the liver, which increases LDL cholesterol clearance from the blood and leads to additional lipid-lowering effects. Depending on the dose and specific statin, cholesterol synthesis is lowered by 10-60%. Statins also decrease the formation of other metabolites of mevalonic acid, such as geranyl pyrophosphate, dolichols, and ubiquinone (coenzyme Q10), which are important in diverse cellular functions resulting in statin toxicity. Statin's use is also associated with unrelated lipid effects such as anti-inflammatory, antithrombotic, inhibition of smooth muscle proliferation, and decreasing platelet aggregation [18,19].

Statins selectively inhibit HMG-CoA reductase, with no considerable affinity for other enzymes or receptor systems, implying that they are unlikely to have pharmacological interactions with other drugs on a pharmacodynamic level. Statins effectively lower plasma LDL concentration [17]. All statins undergo substantial microsomal metabolism via the CYP isoenzyme system in phase I (one) drug metabolism, apart from pravastatin, which is converted enzymatically in the liver cytosol. In humans, 100 CYP genes and 20 CYP proteins have been identified. Most drug metabolism is through CYP proteins; CYP1, CYP2, and CYP3. The CYP system may explain drug-drug interactions by causing interindividual heterogeneity in drug metabolism. Most statins, such as lovastatin, simvastatin, and atorvastatin, are bio-transformed by the CYP3A4 enzyme; however, fluvastatin is largely metabolized by the CYP2C9 enzyme. Rosuvastatin is not substantially metabolized but does interact with the CYP2C9 enzyme [19].

CYP inducers (phenytoin, phenobarbital, barbiturates, rifampin, cyclophosphamide, dexamethasone) decrease the plasma level of statins and their efficacy. On the other hand, CYP inhibitors (cyclosporine, tacrolimus, macrolides, azoles, protease inhibitors) lead to an increase in the plasma level of statins resulting in adverse effects like myositis and rhabdomyolysis [17].

Statins such as lovastatin, simvastatin, and pravastatin are derived from the fungus *Aspergillus terreus*; other statins such as atorvastatin and rosuvastatin are synthetic statins with structures that differ from the fungal-derived statins. The discrepancies in lipophilicity and pharmacokinetic characteristics are attributed to the structural changes of different statins. Highly lipophilic statins are quickly absorbed through the renal tubule walls and do not pass through the urine. As a result, they must be converted to hydrophilic derivatives hepatically and enterically before being excreted from the kidney. Although statins are excreted in small amounts in the urine, the concentration of some statins can double for patients with severe renal failure. Therefore, statins are given in low doses in renal insufficiency, and muscle and hepatic enzyme levels require frequent monitoring [19].

Lowering skeletal muscle chloride channel-one (ClC-1) and related chloride conductance in statin-treated patients may be important in predicting adverse muscle effects. Therefore, it is recommended to limit the use of statins in the elderly and patients with dysfunctional ClC-1 channels, such as myotonic dystrophy [20].

Pharmacogenetics 

Genes regulating statin hepatic absorption have been shown to have the strongest link to genetic variables. The solute carrier organic anion transporter family member 1B1 (SLCO1B1) gene encodes for organic anion transporting polypeptide C (OATP-C) through which statins are transported into liver cells. Except for fluvastatin, most other statins are transported through OATP-C. Fluvastatin easily crosses the hepatocyte membrane due to its lipophilicity. Patients taking pravastatin or atorvastatin who have myopathy had a greater incidence of the OATPC*15 allele [18]. Drug clearance can vary 10-fold due to genetic variations in CYP enzymes resulting in the unpredictability of drug-to-drug interactions in different persons [19].

Statin Usage Frequency

There is an 8% increase (18% to 26%) in the use of statins in > 40 years adults from 2003 to 2012 [21,22]. In 2013, the AHA and ACC revised their guidelines to mitigate cardiovascular complications risk, which significantly expanded the population of patients eligible for statin therapy, especially older age groups [21,23]. As the guidelines were clinically implemented, 12.8 million additional individuals were predicted to qualify for statin therapy, making statins one of the most commonly prescribed medications in the United States [21]. The 2019 AHA/ACC guidelines further supported statins' value (incremental health benefits to net long-term costs) due to their cost-effectiveness and broad spectrum usage across various age strata [9]. One of the reasons suggested behind the increased curative use of statins is their pleiotropic properties, extending their benefits beyond lipid-lowering effects [21,24]. 

The Risk Profile of Patients Using Statin

Statin-associated adverse reactions display significant variability among patients due to different age groups, genetics, body habitus, alcohol use, concomitant drug use, and comorbid conditions [21]. Patients more significantly affected by statin use include [21]: (i) Older people with lower lean muscular mass, multiple drug use, and lessened metabolic function [21,25], (ii) HIV (human immunodeficiency virus) patients with increased drug interaction risk owing to coadministration of anti-viral agents (ritonavir, boceprevir, telaprevir) with statins especially simvastatin and lovastatin [21,26], (iii) People with East Asian ancestry who have higher serum levels of statins such as rosuvastatin than Caucasians [21,27], (iv) Pediatric age group whose dose-exposure-response relationships are poorly defined [21,28], (v) Familial hypercholesterolemia (FH) patients who are drug dependent and are usually on a combination of three to four drugs [21,29], and (vi) chronic kidney disease (CKD) patients who have the impaired capacity to excrete statin [21,30]. Therefore, these diverse patient populations require variable statin types having different pharmacokinetic mechanisms and dosages, or statins are contraindicated [21]. 

Toxicity 

Patients using statins frequently experience muscle pain. Benign muscle pain without substantial biochemical correlations must be distinguished from those with severe myopathies. Statins can cause various skeletal muscle side effects, ranging from muscle pain to muscle cell damage, and they may lead to acute rhabdomyolysis [18,31].

Gastrointestinal symptoms, headaches, and rash are common side effects linked with statin medication, although they are usually minor and transitory. Asymptomatic increases in liver transaminases and myopathy are the most serious side effects. Raised liver transaminases are seen in 1% of patients, and these are related to the dose and individual statin. They are usually seen in the initial three months and require serial follow-up. Myopathy is noticed from a few weeks to more than two years in patients on statin therapy [17].

Myopathies caused by statins include benign muscular pain without CK elevation, myopathy/myositis with considerable CK elevation, fulminant rhabdomyolysis, and HMG-CoA reductase antibody-driven immune-mediated necrotizing myopathy (IMNM). IMNM is associated with symmetrical muscle weakness, which can be acute or subacute. The duration of statin therapy to symptoms varies from two months to 10 years, with an average of three years. Treatment includes discontinuation of statins with immunotherapy [31].

Benefits

The benefits of statins in diabetics have been extensively studied. Statins offer benefits in controlling dyslipidemia associated with diabetes. Furthermore, the beneficial role of statins as primary and secondary prevention in macro and microvascular complications associated with diabetes is well established [32]. 

Although statins may increase the risk of developing T2DM in high-risk individuals, the ADA guidelines recommend continuing statin therapy in these people with regular glucose monitoring as the cardioprotective benefits outweigh the harmful effects [33]. 

Preventative/Treatment Guidelines

The National Institute for Health and Care Excellence (NICE) guidelines (2014) recommend all type 1 DM (T1DM) patients to take statin therapy as primary prevention. Therefore, atorvastatin 20 mg should be offered to patients in >40 year age groups, those having diabetes for >10 years, those having established nephropathy, and those with other risk factors for CVD. It also recommends the usage of atorvastatin 20 mg in T2DM patients with a 10% 10-year CVD risk. In addition, atorvastatin 80 mg should be initiated for patients with pre-existing CVD as a secondary prevention strategy [32].

The 2017 ADA Standards of Medical Care in Diabetes emphasizes the role of lifestyle modifications, such as diet and exercise, in addition to pharmacological interventions in managing diabetes and its complications. ADA recommends that diabetic patients should be treated with a statin, who are 40-75 years old without CVD, 40-75 years old without CVD but with one or more CVD risk factors (LDL-C ≥100 mg/dL, raised blood pressure, chronic kidney disease, albuminuria, smoking, and a family history of premature CVD), >75 years old with or without CVD risk factors, or have a history of CVD regardless of their baseline LDL levels [34]. The new 2023 ADA guidelines suggest initiating moderate-intensity statin in >75 years old diabetic patients only after assessing the benefits to harmful effects or continuing therapy if already on statin [35].

Furthermore, according to the 2023 ADA Standards of Medical Care in Diabetes guidelines, lower LDL goals are recommended for high-risk diabetic individuals, such as <70 mg/dl for patients with high atherosclerotic CVD (ASCVD) risk and <55 mg/dl with ASCVD. High and moderate-intensity statins cause ≥50% and 30-49% reduction in LDL-C, respectively [35].

Statin-induced myopathy

Definition

Statin-induced myopathy covers a wide variety of manifestations occurring as side effects of using statins. It includes various forms of myotoxicity like myalgia, myositis, rhabdomyolysis, and immune and non-immune myopathies [14,36].

Statin myopathy: The American College of Advisory and AHA clinical advisory has defined statin myopathy as any muscle complaints related to statins, such as muscle aches, muscle tenderness, cramps, weakness, fatigue, or CK elevations [14,36].

Myalgia: Myalgia is a mild form of statin myopathy, which is extremely common. It is described as experiencing muscle pains and discomfort, usually involving proximal muscles. It is mostly associated with normal serum CK levels, but it can also present with mild elevation in CK levels, <1000 international units/liter (IU/L) (<5-fold the upper limit of normal) [14,36,37].

Myositis: Statin-associated myositis involves muscle symptoms, such as muscle pain and tenderness with CK elevation (5-10 fold the upper limit of normal). Myositis in statin users demonstrates polymyositis and myolysis in skeletal muscle biopsies [14,36].

Rhabdomyolysis: It is the most severe adverse effect of statin-associated myotoxicity, which has clinically significant effects and can be life-threatening. This syndrome involves myoglobinuria, acute tubular necrosis, azotemia, and metabolic abnormalities, in addition to muscle symptoms. CK levels reach up to >10,000 IU/L, which is >100-fold/ >10 times the upper limit of normality (ULN) [14,36,37].

IMNM: It is an autoimmune myopathy seen in statin users with CK values ranging between 2000 and 20,000 IU/L (10-100 times ULN) [37].

Non-immune mediated myopathy/toxic: The toxic form of statin myopathy does not involve immunomodulatory pathways and has variable presentations. It shows CK levels between 2000 and 20,000 IU/L (10-100 times ULN) [37].

Prevalence/Incidence

The incidence of statin-induced myopathy varies with the type of statin and concomitant drugs used, manifesting a wide range of symptoms. A systematic review of 20 randomized clinical trials and two cohort studies showed that the incidence of rhabdomyolysis was 3.4 per 100,000 person-years in all the statins other than cerivastatin. The incidence of myopathy was higher in patients taking simvastatin, lovastatin, and atorvastatin compared with pravastatin and fluvastatin due to varying metabolic mechanisms. The concomitant use of gemfibrozil with statins increases the myopathy incidence ten times more than statin alone [38]. 

A narrative review on statin-related myopathy demonstrated that the incidence of myalgia is 10% as compared to rhabdomyolysis, which is rare, in persons taking statins [11]. Furthermore, among all the side effects of statins, statin-associated myopathy was reported in 6-25% of patients in a database analytical study [39].

A cross-sectional study of 300 patients showed the prevalence of myalgia in 51% of patients. It also exhibited a statistically significant relation of myalgia to the type (p=0.05), dose (p=0.031), and duration of statin (p=0.036) used [40].

Risk Factors

The risk factors associated with myopathy, which developed after the use of statins, are manifold. The predisposing risk factors include age, gender, ethnicity, drug interactions, and comorbid conditions. A cohort study determined a three-fold and six-fold increase in the risk of moderate/severe myopathy in new statin users in women and men, respectively. Regarding ethnicity, Caribbean and Black Africans have the highest likelihood of developing statin-related myopathy compared to White individuals. It is also confirmed that different comorbid conditions are correlated with statin-induced muscle symptoms (SAMS), such as T1DM, hypothyroidism, treated hypertension, and chronic liver disease, especially in females [41].

A study reported evidence of rhabdomyolysis in patients taking cerivastatin and gemfibrozil combination therapy, which led to the discontinuation of cerivastatin from the market by the United States Food and Drug Administration (FDA) [36]. In addition, the Prediction of Muscular Risk in Observational Conditions (PRIMO) study, an observational study conducted in a primary care setting in France, also supported the fact that the major risk factors of myopathy resulting from high-dose statin usage is the personal or family history of muscle symptoms, hypothyroidism, and elevated CK levels [42]. 

Another study added the patient-related risk factors, such as low BMI, renal/hepatic dysfunction, statin-CYP inhibitors concomitant use, albumin, and alpha-1 glycoprotein changes as well as the dosage of statins used. It’s also proposed that lipophilic statins (simvastatin) induce an exaggerated risk of myopathy versus hydrophilic statins (pravastatin) because of the different mechanisms of action [43].

A population-based cohort study validated risk algorithms to evaluate the risks of four clinical outcomes associated with statin use by applying the Q-Research validation cohort and The Health Information Network (THIN) validation cohorts. Their statistics from the THIN cohort implied that the algorithm explains 42.15% of the variation for moderate to severe myopathy. This algorithm will help to identify high-risk individuals [41]. Table 1 summarizes the risk factors responsible for myotoxicity in patients receiving statin therapy [36,41-43].

**Table 1:** Risk factors of statin-induced myopathy SLCO1B1: Solute Carrier Organic Anion Transporter Family Member 1B1; CYP: Cytochrome P450; HIV: Human Immunodeficiency Virus [36,41-43]. Table created by Sara Tariq (author)

Patient-Related Risk Factors
Age (80 Years or Older)
Gender (Mostly Females)
Ethnicity (Caribbean or Black African)
Low Basal Metabolic Index
Family or Personal History of Statin Intolerance or Muscle Symptoms
Genetics (SLCO1B1 or CYP450 Gene Polymorphisms)
Heavy Alcohol Consumption (>2 Alcoholic Drinks/Day)
Vitamin D3 Deficiency (levels <15mg/ml)
Strenuous Exercise
Multiple Drug Regimens
Comorbidities (Hypothyroidism, Diabetes Mellitus Type 1 or 2, Hypertension, HIV, Liver, and Kidney Disease)
Major Trauma or Surgery
Drug-Related Risk Factors
Dose/Intensity of Statin (High or Moderate-Intensity > Low-Intensity)
Type of Statin (Simvastatin > Fluvastatin)
Duration of Use of Statin
Metabolic Mechanism of Statin (Simvastatin, Lovastatin, Atorvastatin > Pravastatin, Fluvastatin)
Lipophilicity vs. Hydrophilicity of Statin (Simvastatin > Pravastatin)
Drug Interactions (CYP Inhibitors, such as Antibiotics, Antidepressants, Antiarrhythmics, etc.)

Clinical Spectrum

Many studies elaborated on the clinical spectrum observed in patients developing myopathy after statin use. Different clinical signs and symptoms can appear as a result of taking statins, ranging from muscle pain and cramps to dark urine and renal insufficiency. Variable presentations are noted in statin-related myopathies, such as myalgia with or without CK elevations, muscle weakness, muscle tenderness or myositis, exercise intolerance, fatigability, or persistent myalgia and elevated CK levels after statin withdrawal [14,36,44,45].

Another study described the range of statin-induced muscle symptoms from clinically benign myalgia to rare but life-threatening rhabdomyolysis as well as toxic and immune-mediated, severe necrotizing myopathies [11,46]. A study conducted in 2016 described statin-associated muscle symptoms (SAMS) as the most frequent of all the side effects, with myalgia affecting 5-10% of statin users [47]. It also narrates that rhabdomyolysis and statin-induced necrotizing autoimmune myopathy (SINAM) are rare [47,48].

Pathophysiology/Causes

Multiple mechanisms have been presented to elucidate the pathophysiology of statin-related myotoxicity, but the exact mechanism is not known to date. Various mechanisms involved include muscle excitability, mitochondrial dysfunction, ubiquinone (coenzyme Q10) depletion, calcium homeostasis impairment, apoptosis induction, genetic determinants, and immunologic and non-immunologic parameters. Most of the mechanisms of statin-associated myotonic symptoms appear to be interlinked [36,44].

Muscle excitability: HMG-CoA reductase inhibitors or statins are responsible for inhibiting the conversion of HMG-COA to mevalonate, decreasing posttranscriptional lipid modifications (prenylation) of certain proteins such as transfer-ribonucleic acid (tRNA), glycoproteins, and electron transport chain (ETC) proteins like heme A, coenzyme Q10, and small G-proteins. The inactivation of GTPases, especially Rab, resulting in defective intracellular vesicular trafficking, is critical in myotoxicity related to statins [36,44,49].

ClC-1 channels stabilize the sarcolemma, lowering these channels induced hyperexcitability leading to muscle cramps and myalgia. In addition, increased activity of the protein kinase C theta (PKC theta) isoform, which might inactivate ClC-1, may contribute to sarcolemma excitability disruption, resulting in statin-induced myopathy [20].

Mitochondrial dysfunction and ubiquinone (coenzyme Q10) depletion: Statins directly affect the mitochondrial respiratory chain in addition to reduced cholesterol synthesis. Coenzyme Q10, a cofactor of ETC and antioxidant in mitochondria and lipid membranes, gets depleted, leading to impairment in cellular respiration and causing muscle-related toxicities [36,44].

A systematic review was conducted to determine the relation of coenzyme Q10 levels with statin administration. It showed reduced levels of coenzyme Q10 in 16 patients receiving simvastatin 80 mg/day [36,44,50]. A case report by Thomas et al. indicated that the prescription of statin uncovered mitochondrial encephalopathy, lactic acidosis, and stroke-like episode (MELAS) syndrome in genetically susceptible patients due to reduced coenzyme Q10 levels [51].

Calcium homeostasis impairment: Lorkowska et al. found that statin increases calcium levels in the endothelium of vascular smooth muscle cells (VSMC) by reducing isoprenoids, which inhibits the L-type calcium (Ca2+) channels [36,44,52]. Other mechanisms revealing the increased concentration of intracellular calcium include calcium release from mitochondria and decreased sarcoplasmic reticulum calcium ATPase (SERCA) activity [36,44].

A study revealed that lovastatin causes a decreased density of sodium-potassium (Na+-K+) ATPase pumps in skeletal and cardiac muscle sarcolemma, which results in an increase in sarcoplasmic calcium concentration via sodium-calcium (Na+-Ca2+) antiport [44,53].

Apoptosis induction: Apoptosis of skeletal or smooth muscle cells resulting from statin usage is linked with other pathophysiologic pathways. Statin causes a diminished translocation of Ras homolog family member A (Rho-A) and Ras-related C3 botulinum toxin substrate 1 (Rac-1) from the cytosol to the membrane. Statins also affect the protein synthesis mechanisms via a decrease in isoprenoids (lowers the protein geranylgeranylation and/or farnesylation), ultimately leading to increased cytosolic calcium levels and activation of the mitochondrial apoptotic cascade. Moreover, a decrease in coenzyme Q10 also results in the apoptosis of muscle cells [36,44,54].

Genetic determinants: Polymorphisms in genes encoding uridine diphosphate (UDP)-glucuronosyl-transferase-1 (UGT1) affect the metabolic derivatives of statin in phase॥ (two) of metabolism in the biotransformation pathway. Another genetic variation associated with statin-induced myotoxicity is the polymorphisms in the solute carrier organic anion (SLCO) family of membrane transporters [55].

The Statin Response Examined by Genetic Haplotype Markers (STRENGTH) study stated that the several variants in the SLCO1B1 gene, which encodes OATP1B1, affect the hepatic uptake of statins leading to myopathy [56-58].

Immunologic: The statin-associated necrotizing myopathy mostly affects males and causes proximal muscle weakness with severe CK elevations [59]. Statins can contribute to the increased anti-HMG-CoA reductase expression forming neoantigens, or they can make HMG-CoA reductase more immunogenic by modifying protein conformation. These mechanisms break the immune tolerance and cause anti-HMG-CoA reductase myopathy, a subtype of immune-mediated necrotizing myopathy associated with statin use [60].

Toxic: Statin is one of the most common medications causing toxic myopathies [61]. Toxic statin myopathy is a non-immune mediated myopathy with variable symptoms and negative anti-HMG-CoA reductase antibodies. Pathologic findings show necrotic and regenerating muscle fibers expressing major histocompatibility complex (MHC) class I, similar to IMNM [37].

Grable-Esposito et al. mentioned in a report that 25 IMNM patients from two neuromuscular centers showed persistent symptoms of muscle weakness even after discontinuing statin or had a relapse after initial improvement from immunosuppressant drugs [62].

**Figure 1:**
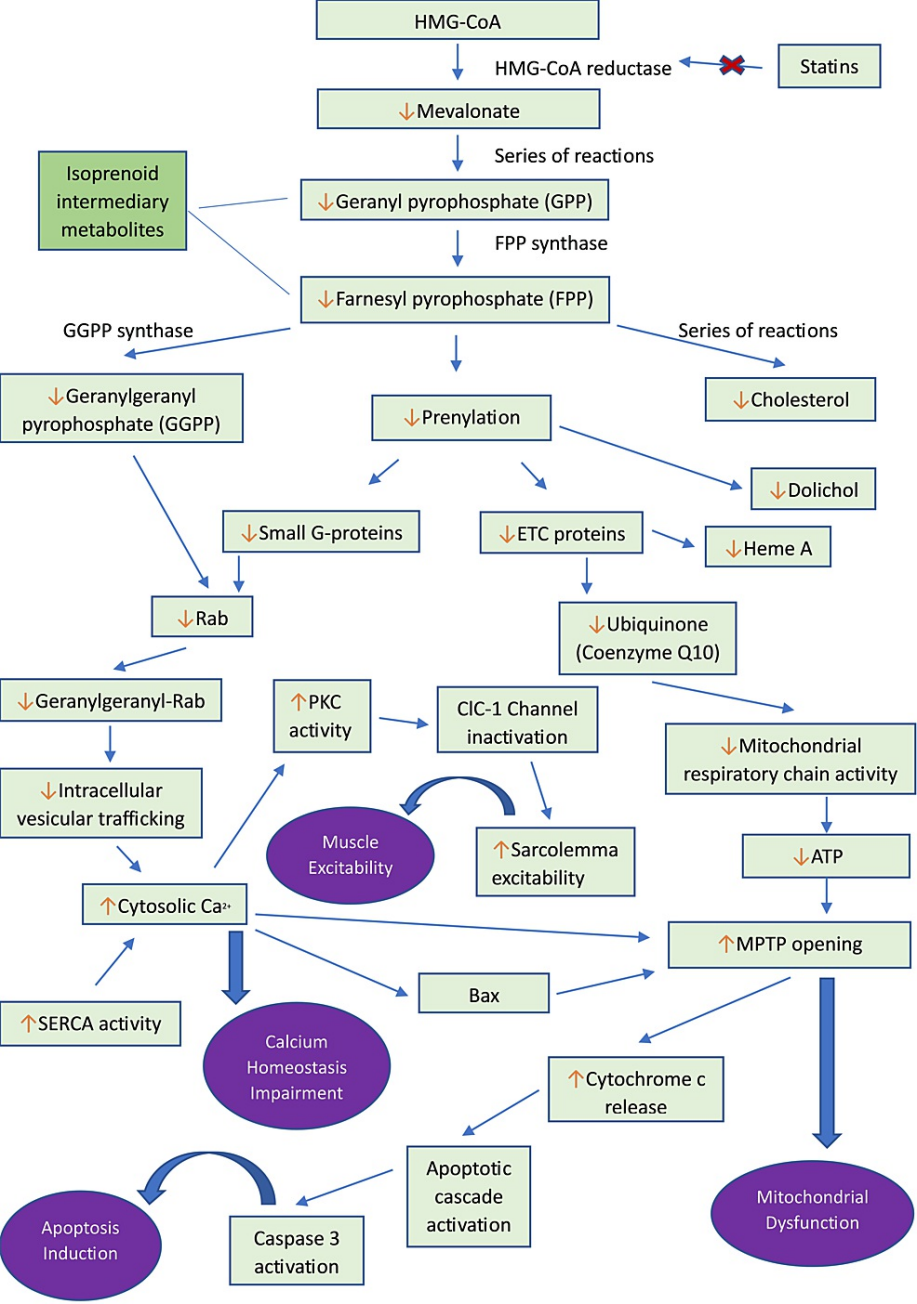
Pathophysiology of statin-induced myopathy HMG-CoA: 3-Hydroxy-3-Methylglutaryl Coenzyme A; ETC: Electron Transport Chain; Rab: Ras-Associated Binding Protein; PKC: Protein Kinase-C; ClC-1: Chloride Channel-1; SERCA: Sarcoendoplasmic Reticulum Calcium ATPase; ATP: Adenosine Triphosphate; MPTP: Mitochondrial Permeability Transition Pore, Bax: Bcl-2 Associated X Protein [36]. Figure credit: Sara Tariq (author)

Figure 1 summarizes the pathophysiology of statin-induced myopathy [36].

Diagnosis

Patients experiencing side effects from statins are diagnosed by measuring CK levels in the blood, a marker of muscle damage [1]. The National Lipid Association has presented diagnostic strategies for statin-induced myopathy by providing consensus guidelines to healthcare professionals regarding the safe use of statins. These recommendations advised health professionals to rule out other causes of muscle symptoms and CK elevations initially in patients receiving statin therapy instead of diagnosing statin-related myopathy directly [39,63]. Table 2 highlights when to order CK levels in symptomatic and asymptomatic patients taking statins for diagnosing statin-induced myopathy [39,63].

**Table 2:** Diagnostic strategy of statin-induced myopathy in patients receiving statins Note: Patients receiving statins can develop intolerable symptoms with or without elevation in CK levels, so close monitoring of symptoms is also necessary [39,63]. CK: creatine kinase Table created by Sara Tariq (author)

Patients receiving statin therapy	Diagnosis of statin-induced myopathy
Asymptomatic patients	No need to order CK levels
High-risk patients (old age, diabetic, renal/hepatic dysfunction, concomitant medications causing myotoxicity)	Ordering CK levels can be considered but not routinely necessary
Symptomatic patients (having muscle aches, muscle weakness, muscle tenderness)	Order CK levels

Toxic statin myopathy requires muscle biopsy in addition to CK levels if the clinical symptoms do not improve after four to six weeks of statin discontinuation. For IMNM patients, anti-HMG-CoA reductase antibodies should be ordered. The techniques used for detecting anti-HMG-CoA reductase antibodies include observing a characteristic HMG-CoA reductase-associated liver indirect immunofluorescence pattern (HALIP) in tissue sections and confirming them with enzyme-linked immunosorbent assay (ELISA) or blotting later on [37].

Statin-induced myopathy in diabetic patients

Prevalence

From 2004 onwards, according to multiple studies on the subject, the American College of Physicians (ACP), the ADA, theNational Cholesterol Education Program Adult Treatment Panel III-Revised (NCEP-ATP III-R), and other well-known medical societies have agreed on the utility of statin therapy for almost every diabetic patient [64-66]. Nowadays, every patient with diabetes aged greater than 40 years should be prescribed a statin agent even with the absence of cardiovascular risk factors, according to the ADA [34]. Hence, with the expanding population benefiting from HMG-CoA reductase inhibitors exacerbated by the rising rate of diabetes, we are faced with a significant number of reported SAMS, especially in diabetic patients.

Amongst other risk factors of statin-induced myopathy, multiple studies have identified diabetes as a major one. A meta-analysis including 44 studies demonstrated the association of DM and myopathy in patients taking statins (OR 2.34, 95%CI 2.06-2.64). To a lower extent, in the same study, diabetes was also identified as a risk factor for rhabdomyolysis (OR 1.96, 95%CI 1.79-2.15) [67]. Manoj et al. found that diabetic patients on atorvastatin had a 7.5% prevalence of myopathy [68].

A study conducted in a military hospital in Pakistan concluded that 8.4% of atorvastatin-treated patients developed myopathy. Muscular pain was the most common symptom, with 93% of patients reporting it. It was evenly disseminated across the upper and lower limbs. Otherwise, there were no complaints of stiffness. Symptoms were experienced seven days after the initiation of atorvastatin, with a median time of 90 days [69].

Lastly, a cohort study conducted on 32,225 members of the Health Maintenance Organization (HMO), comparing the prevalence of myopathy in diabetic and non-diabetic patients taking or not taking statins, showed that the prevalence rate of myopathy was 18.9 per 1000 person-years (95% CI 17.3-20.7) in diabetic patients not taking statins in comparison to 12.6 per 1000 person-years (95%CI 11.6-13.7) in non-diabetic patients not taking statins. More importantly, the use of statins in diabetic patients was linked to a 29% increased incidence of myopathic incidents (hazard ratio (HR) = 1.29; 95%CI 1.15-1.45; P < 0.001). It is worth mentioning that patients on statins compared to the control group were older and had a higher BMI, high blood pressure, high-risk lipid profiles, and more comorbidities. Additionally, they were more likely to be on additional pharmaceutical drugs [70].

The finding of statin use in diabetic patients was not only associated with SAMS but was responsible for doubling the risk of myopathic adverse effects. Nevertheless, most of the myopathic events occurred in the absence of CK elevations or at clinically insignificant levels of CK elevation. The adverse effect of severe myositis or rhabdomyolysis occurred at a rate of less than one per 1000 person-years, compared to a rate of 24-27 per 1000 person-years for other SAMS [70].

Variables Associated With Statin-Induced Myopathy

Increased statin regimen doses and duration have been linked to a higher incidence of statin-induced myopathy in diabetic patients. In the Study of the Effectiveness of Additional Reductions in Cholesterol and Homocysteine (SEARCH) trial, subjects with DM on higher doses of simvastatin (80 mg per day) were associated with increased “definite or incipient myopathy” after the first year (risk ratio (RR) of 1.7 (95%CI 1.0-2.9) and RR of 2.3 (95%CI 1.1-4.9)) [58]. The large observational study PRIMO also reached the same conclusion, as it was proved that 14.9% of patients taking higher doses of atorvastatin (40-80 mg a day) suffered from myopathy [42]. In the previously discussed study conducted in a military hospital in Pakistan, the frequency of myopathy climbed as the duration of atorvastatin treatment increased (p-value < 0.05) [69].

Myopathic occurrences in diabetic individuals were also predicted by age, simultaneous use of fibrates or corticosteroids, and BMI. Sulfonylurea use was associated with a 17% higher risk (HR = 1.17; 95%CI, 1.03-1.33; P = 0.015); however, metformin use was associated with a lower risk of myopathy (HR = 0.79; 95%CI, 0.69-0.90; P = 0.001). Neither the usage of insulin nor the use of thiazolidinediones was found to be a risk factor. Glycemic control was similarly unrelated [70].

After a warning on a pharmacovigilance safety issue, a recent study demonstrated an increase in occurrences of myopathy when DPP-4 inhibitors and statins were used together (adjusted reporting OR (ROR)= 4.5 (2.7-7.6)). However, the occurrence of myopathy was lower than when statins were used alone (adjusted ROR=6.2 (5.7-6.8)), which supported that the additive model did not indicate a probable drug-drug interaction [71].

This should raise caution when prescribing statins in diabetic patients who are elderly, overweight, on fibrates, corticosteroids, calcium-channel blockers, or other diabetic regimens, even when the benefit of statin prescription largely surpasses the risk of myopathy, which was started by the ACC, the AHA, and the National Heart, Lung, and Blood Institute (NHLBI) clinical advisory on the use and safety of statins [72].

Pathophysiology/Causes

Reduced mevalonate pathway products, mitochondrial malfunction, variations in gene expression related to apoptosis and protein degradation, and genetic predisposition have all been defined as contributing factors to statin-induced myopathy [73]. 

Gene expression may provide plausible explanations for the changes that occur on the molecular level of myocytes, which is clinically expressed as myopathy in diabetic patients on statins. To our knowledge, very few studies were conducted for this purpose until the SEARCH study on gene association analysis could demonstrate the strong correlation of SLCO1B1 gene variants with the occurrence of myopathy in simvastatin users. Precisely speaking, the presence of single nucleotide polymorphisms (SNP) within the SLCO1B1 gene on chromosome 12 was identified as the culprit. SLCO1B1 is expressed in cells and regulates statins’ hepatic uptake [58]. Moreover, SLCO1B1 variants were associated with severe forms of myopathy and rhabdomyolysis with levels of CK >10x ULN. In addition, this correlation was described as dose-related, emphasizing the significance of dose-genotype interaction [74].

Since this variant is not linked to atorvastatin-induced myopathy, which is less lipophilic than simvastatin, it's thought that the effects of SLCO1B1 are dependent on the statin type. As an explanation, lipophilic statins may be susceptible to non-selective diffusion into extra-hepatic tissues, including skeletal muscle cells. In contrast, hydrophilic statins may be actively carried into hepatocytes expressing the organic-anion-transporting polypeptides (OATP) transporter [75]. In the same context, according to the PRIMO trial, the most hydrophilic statins, pravastatin, and fluvastatin, are associated with the lowest incidence of myalgia, but simvastatin (the most lipophilic statin) is most likely to induce SAMS [42].

The Genetics of Diabetes Audit and Research (Go-DARTS) study tested the association of rs4149056 (Val174Ala) and rs2306283 (Asp130Asn), which are common variants of the SLCO1B1 genome, with statin intolerance in 4196 T2DM patients. Many factors contributed to statin intolerance, defined in the study as abnormal CK or alanine aminotransferase (ALT) levels, including both genotypes, statin medication compliance, duration of therapy, statin maximum doses, the association of other lipid-regulating drugs, cytochrome P450 (CYP) 3A4 inhibiting drugs, and age [76]. 

Moreover, the presence of the Ala174 genome was associated with a lower LDL response, which the discontinuation of treatment could explain due to adverse effects. As a result, diabetic patients with SLCO1B1 gene variants were more susceptible to adverse cardiovascular events [76]. The Clinical Pharmacogenetics Implementation Consortium Guidelines suggest detailed recommendations to tailor statin therapy according to SLCO1B1 to reduce myopathic incidents, highlighting rosuvastatin 20 mg as high-intensity, rosuvastatin 5-10 mg as moderate-intensity, and fluvastatin 20-40 mg as low-intensity statin carrying lowest SAMS risk [77].

Other factors incriminated in the pathogenesis of DM were associated with statin-induced myopathy. For example, GLUT4 mRNA reduction was related to statin-induced myotoxicity exacerbated by aging. The principal characterization of diabetes, which is insulin deficiency, has also been associated with SAMS [20,78]. Lastly, opening an ATP-sensitive potassium channel (KATP channel), which inhibits insulin secretion, could induce myopathy in diabetic patients under a statin regimen [79].

The studies involved in the research and explanation of the mechanisms responsible for increased statin-associated myositis in diabetic patients are counted on hand fingers. It should raise awareness to encourage additional studies on this subject, as understanding the pathophysiological causes behind it would lead to the adaptation of statin therapy in diabetic individuals according to the identification of specific markers.

Prognosis of diabetic patients taking statins

One of the key features associated with DM is hyperlipidemia, endothelial dysfunction, and inflammation, which contribute to atherosclerosis and increases the overall cardiovascular risk [80]. Therefore, statins are a must to address atherosclerosis in the treatment regimen of diabetic patients.

Besides its side effects, statins significantly improve lipid profile and endothelial function and have great anti-inflammatory and antithrombotic properties [60].

In a randomized, double-blind trial conducted by Werida et al. regarding the effect of atorvastatin versus rosuvastatin on inflammatory biomarkers, their results showed that compared to baseline, the use of atorvastatin and rosuvastatin were associated with a considerable decrease in serum levels of C-reactive protein (CRP), sortilin, leptin, and increase of adiponectin levels [81]. Thus, the respective change in each of these biomarkers significantly contributes to improving the atherogenic index and managing cardiovascular risk. Moreover, cardiac insulin resistance, oxidative stress, inflammation, and autonomic neuropathy associated with diabetes contribute to diastolic and systolic dysfunction in diabetic patients [82]. 

However, the data are very polarized regarding the effect of statins on myocardial function. A study conducted by Rubinstein et al. regarding myocardial function evaluation in patients taking statins showed a decrease in diastolic function measured by strain imaging and advanced echocardiographic imaging with tissue Doppler in patients with no history of cardiovascular events [83]. On the other hand, a study conducted by Mizuguchi et al. showed net amelioration of carotid arterial stiffness as well as myocardial function in patients with preserved left ventricular ejection fraction [84].

Generally, the mortality rates from cardiovascular causes for diabetic patients have not decreased significantly compared to non-diabetic patients due to less reduction in risk factors, heart disease incidence, and less benefit from medical treatments comparatively [85]. A study by Yun et al. demonstrated insulin resistance, hypoglycemia, and genetic risk factors to be the culprits of CVD risk in diabetic patients. They recommended intensive lifestyle modification, sodium-glucose cotransporter-2 (SGLT2) inhibitors, and glucagon-like peptide-1 (GLP-1) agonists for CVD management in diabetic patients [86].

Therefore, the prognostic value of statins in diabetics is incumbent upon risk factors and management strategies and needs further exploration through promising literature reviews. An individualized management approach for diabetic patients should be implemented, considering all the characteristics or risk factors associated with the patient.

Monitoring and management of myopathy in patients taking statins

The algorithm to manage a statin-induced myopathy requires considering a series of factors, which include reassessing statins' benefit-to-harm ratio, establishing a preliminary diagnosis, ruling out the contributing entities, reassuring the patient, and changing statin treatment regimen by modifying type, dosage, and frequency [47].

Different guidelines have yet to reach a consensus for managing statin-related myopathy. Nonetheless, all the guidelines agree on ordering diagnostic tests based on myopathic symptoms experienced by the patients. Checking serum CK levels is recommended if a patient experiences muscular side effects [68]. Furthermore, the amount of CK elevation also guides therapy. For example, if the serum CK levels rise > 10x ULN along with symptoms, statins should be discontinued to avoid aggravating symptoms [72]. However, the consensus of different guidelines varies regarding serum CK levels ranging from three to 10 times ULN. They recommend stopping statin unless the patient reports symptom improvement [68]. 

Initial Evaluation

It is essential to exclude potentially contributing factors that enhance the myopathy caused by statin. Some of the contributing factors identified require an assessment of thyroid function, vitamin D levels, and concomitant drug use. Thyroid dysfunction, such as subclinical hypothyroidism, also has implications in statin-related myopathy. Similarly, vitamin D levels <15 mg/dl can result in myalgia in statin users [87]. Furthermore, treating vitamin D deficiency may enhance and reestablish statin tolerance in patients previously suffering from myopathy [88]. Ruling out the coadministration of CYP3A4 and CYP2C9 inhibitors with statins is also crucial to preventing myopathy [87].

Statin Therapy

As statins have proven effective in reducing cardiovascular morbidity and mortality, a statin-based approach is critically important for patients with high-risk profiles. Different approaches can be implemented for managing statin-induced myopathy, such as changing the statin type, dosage, and frequency or reusing the same statin [87]. 

Switching statins: The strategy of switching statins can be beneficial. A study by Hansen et al. investigated the outcomes related to statin-induced myopathy in 45 patients. Thirty-seven patients were commenced on a different statin after developing myopathy from statin use. Recurrent symptoms do not develop in 43% of these patients, implying their tolerance to the new statin therapy [89]. Therefore, statins carrying a lower risk of myopathy (fluvastatin or pravastatin) may be considered in statin-intolerant patients, according to PRIMO trial results [42].

Changing the dose or frequency of statin: Another statin-based approach for statin-intolerant patients involves using a lower dose (de-challenge) or less frequent dosing of a long-acting statin, especially rosuvastatin. Rosuvastatin may be used one to three times a week or ≤10 mg every other day in a statin-intolerant patient when indicated. Backes et al. retrospectively analyzed supplementation of alternate-day rosuvastatin in 51 patients with statin intolerance. Thirty-seven patients (72.5%) tolerated its use, reducing LDL-C levels by 34.5% (p < 0.001) [90].

Non-Statin Therapy

Statin intolerance or inability to effectively reduce LDL-C levels after maximally tolerated statin dose mandates the use of non-statin therapies. The well-tolerated and commonly used non-statin drug is ezetimibe. A study conducted by Stein et al. in 2008 assessed the effectiveness of fluvastatin XL (extended-release) alone or combined with ezetimibe in statin-intolerant patients with muscular side effects. They found that adding fluvastatin XL 80 mg with ezetimibe or using ezetimibe alone established tolerance in previously intolerant patients and reduced LDL-C by 15% [91].

Fibrate therapy has a proven CVD benefit established in hypertriglyceridemia patients, causing an approximate 15% reduction of LDL. Therefore, resins or fibrates may be added to ezetimibe if ezetimibe alone is insufficient to reduce LDL levels to the desired target. However, It is advisable to use fenofibrate with fewer statin interactions when indicated, rather than gemfibrozil, notorious for causing myopathy [45].

In the Lipid Research Clinics Study Coronary Primary Prevention (LRC-CPPT) Trial, bile-acid sequestrants (BAS) were considered another therapy to help reduce cardiovascular events by lowering cholesterol absorption [92]. In addition, the concomitant use of ezetimibe and BAS can reduce LDL-C even greater [93].

Niacin alone decreased CVD mortality and morbidity in the Coronary Drug Project study, as it causes a 20% reduction in LDL-C if given every day with a dose ranging between 500 mg and 2000 mg [94]. In addition, niacin can be added to ezetimibe and BAS for a greater reduction of LDL-C in patients developing statin intolerance [95]. However, patients using niacin can experience flushing, the main adverse effect leading to drug discontinuation in 25% patient population [96]. 

The human monoclonal antibodies to proprotein convertase subtilisin/kexin type 9 (PCSK9), alirocumab, and evolocumab [47], have been approved for use in the United States and Europe for statin-intolerant patients. Both drugs provide cardiovascular benefits by reducing LDL-C by >50% [45].

The Goal Achievement After Utilizing an Anti-PCSK9 Antibody in Statin-Intolerant Subjects-2 (GAUSS-2) randomized trial compared evolocumab and ezetimibe in statin-intolerant subjects to achieve a target LDL goal. As a result, evolocumab does not cause myopathy in addition to a greater reduction of LDL compared to ezetimibe [97].

Management of Rhabdomyolysis and IMNM

Rhabdomyolysis requires careful monitoring and supportive care, including adequate hydration to facilitate renal excretion of myoglobin, nearly always in an in-patient setting [49]. The European Neuromuscular Centre (ENMC) has recommended initiating corticosteroids plus methotrexate for IMNM patients. Alternative medications used in place of methotrexate include mycophenolate, azathioprine, cyclosporine, cyclophosphamide, and tacrolimus [60]. The ENMC treatment guidelines further suggest using rituximab for steroid non-responsive anti-signal recognition particle (anti-SRP) patients [98]. Additionally, intravenous immunoglobulins are recommended for refractory disease in anti-HMG-CoA reductase myopathy patients [60].

*Monitoring* 

Statin therapy requires regular monitoring on initiation or dosage increase of statin. Monitoring includes observing the manifestation of muscular symptoms such as myalgia, tenderness, or weakness. Serum CK levels must be ordered when symptoms manifest. It is recommended to closely monitor high-risk patients with multiple statin myopathy-related risk factors as they may be susceptible to developing rhabdomyolysis [99]. 

The moderate (3x-10x ULN) serum CK levels rise requires weekly monitoring and specialist advice, whereas the severe (>10x ULN) mandates discontinuation of statin [99].

Checking serum CK levels on initiating statins gives a standard baseline [99]; however, routine monitoring of serum CK levels is not recommended unless therapy modifies or the patient acquires comorbidity [99,100]. CK is not always associated with myopathy, and many other factors can elevate CK, such as exercise, drug interactions, genetic variants, CoQ10 deficiency, and vitamin D deficiency [101]. Several research groups have concluded that regular screening and monitoring of CK levels and surveillance of liver function tests are not cost-effective methods to identify the presence of myopathy [102].

## Conclusions

Our review article concluded that dyslipidemia is one of the main predisposing cardiovascular risk factors contributing to increased atherogenic index in diabetic patients. Statins are recommended to reduce cardiovascular risk and mortality owing to their lipid-lowering and pleiotropic effects. On the contrary, their use gets limited due to myopathic side-effects leading to drug non-adherence and discontinuation. The pathophysiology of statin-induced myopathy in diabetic patients depends on the patient and drug-related risk factors. The drug-related risk factors implicated in statin-induced myopathic events include lipophilicity, dose, duration, type, metabolism, and drug-drug interactions. In addition, age, gender, ethnicity, genetics, and comorbidities constitute the other risk factors associated with patients experiencing myopathic side effects from statins. Furthermore, DM is itself a risk factor in causing statin-induced myopathy. Therefore, employing a comprehensive patient-centered approach by assessing contributing risk factors, monitoring CK levels, and using alternate lipid-lowering therapy is significant for managing myopathy in diabetic patients taking statins. The prognosis of statins in diabetics requires further evidence considering the complex nature of risk factors and disease process.
